# A nonossifying fibroma of the femoral neck treated with curettage and dynamic hip screw with free fibular bone graft: a case report

**DOI:** 10.1097/MS9.0000000000001451

**Published:** 2023-11-16

**Authors:** Mohamad Al Masri, Hussain Muhammed, Ola Mahmoud Alrezej, Ameen Esa Alfrouh

**Affiliations:** Damascus university Faculty of Medicine

**Keywords:** case report, free fibular bone graft, intracapsular neck of femur, nonossifying fibroma, pathologic fracture

## Abstract

**Introduction and importance:**

Nonossifying fibroma (NOF) is a benign fibrogenic lesion that is related to dysfunctional ossification and one of the most common benign bone tumors in childhood with incidence rate of 30–40% of skeletally immature children.

**Presentation of case:**

A 25-year-old female with left hip pain for the past 2 months, which was treated conservatively, presented with severe pain in the hip. X-ray showed a pathologic fracture in the neck of the femur with underlying lesion. MRI showed an osteolytic lesion in neck of the femur. Curettage of the lesion was done with free fibular bone graft and stabilized by Dynamic Hip Screw and specimen sent to pathology. The pathology report consisted with NOF. No-weight bearing for 8 weeks with physical rehabilitation were ordered and six months later the patient had a full range of motion and healed fracture.

**Clinical discussion\Conclusion:**

This study revealed that the surgical treatment with bone graft for pathologic femoral neck fracture and underlying NOF allowed a quick return to mobility and can be fixed sufficiently to achieve excellent postoperative recovery.

Highlights

- Non-ossifying fibroma (NOF) is a benign fibrogenic lesion that is related to dysfunctional ossification and one of the most common benign bone tumors in childhood with incidence rate of 30–40% of skeletally immature children.
- A 25- year- old female with left hip pain for the past 2 months, which was treated conservatively, presented with severe pain in the hip. X-ray showed a pathologic fracture in the nick of the femur with underlying lesion.
- MRI showed an osteolytic lesion in neck of the femur. Curettage of the lesion was done with free fibular bone graft and stabilized by Dynamic Hip Screw (DHS) and specimen sent to pathology. The pathology report consisted with NOF. No-weight bearing for 8 weeks with physical rehabilitation were ordered and six months later the patient had a full range of motion and healed fracture.
- This study revealed that the surgical treatment with bone graft for pathologic femoral neck fracture and underlying NOF allowed a quick return to mobility and can be fixed sufficiently even in the absence of microscopic surgery techniques to achieve excellent postoperative recovery.

## Introduction

Nonossifying fibromas (NOFs) are benign bone tumors accounting for 2% of all primary bone tumors typically occurring during the second decade of life with male predominance. They were first described by Jaffe and Lichtenstein in 1942^1^. NOFs are commonly located at the distal femur, distal tibia, and proximal tibia. Etiologically, they are believed to arise from bone marrow cell lineage or the physis, making them more of a developmental bone defect^2^. NOFs are usually asymptomatic, with spontaneous regression by 20–25 years, but can also result in pathologic fractures if the lesion involves more than 50% of the bone diameter^3^. They are typically diagnosed incidentally on plain radiographs, appearing as cortical osteolytic lesions with sclerotic margin^4^. The standard treatment is curettage and cancellous bone graft. In cases of impending fracture or fracture, bony fixation or even arthroplasty is required^5,6^.

In this case, we are focusing on the rare and challenging location, the femoral neck, and emphasize the success of curettage and free fibular bone grafting in the absence of microsurgery availability.

## Presentation of case

This case report adheres the Surgical Case Report (SCARE) 2020 standards^7^.

A 25-year-old female presented to our emergency department complaining of sudden onset severe pain in her left hip and an inability to walk without trauma. She had experienced insidious and dull aching pain in the same hip for the past 2 months, which was initially treated conservatively.

The physical examination revealed an externally rotated left limb, an inability to check the range of motion due to severe pain, and tenderness presented on pressure over the lift groin. There were no skin bruises, wounds, or open fractures. There is no history of fever, anorexia or weight loss, or any other diseases. No other concomitant injuries were detected, and vital signs were normal.

**Figure 1:**
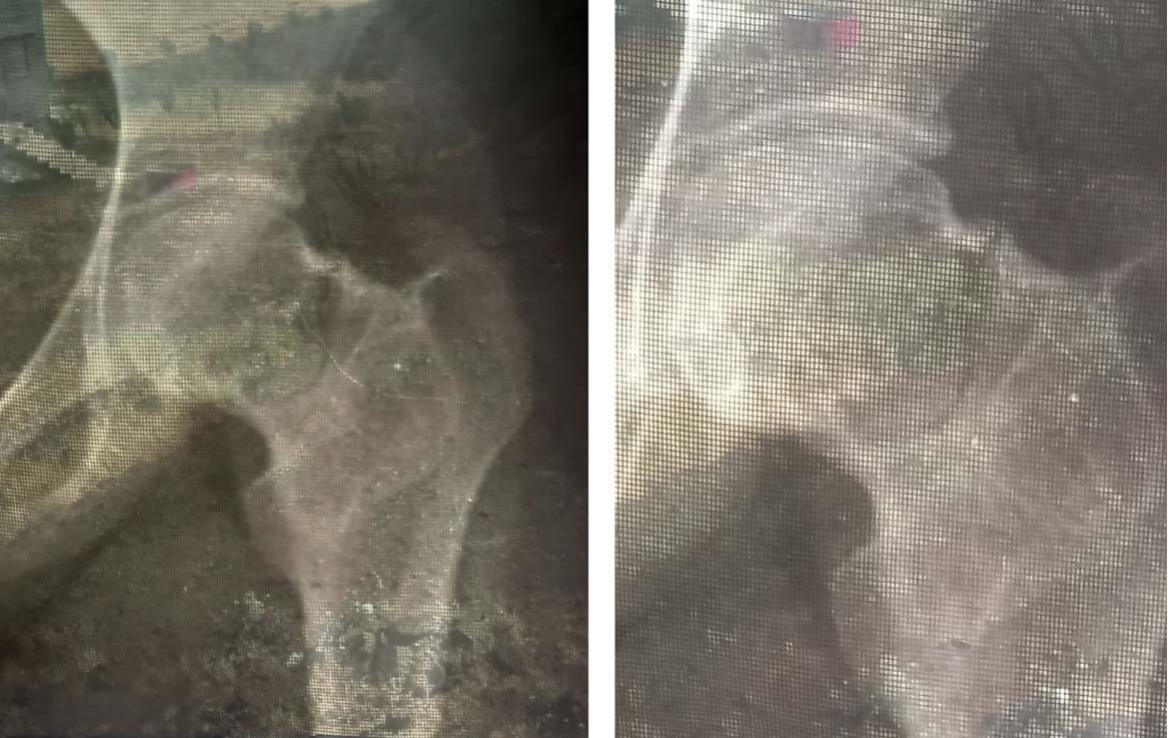
AP X-ray showing intracapsular neck of femur fracture with underlying lesion.

**Figure 2:**
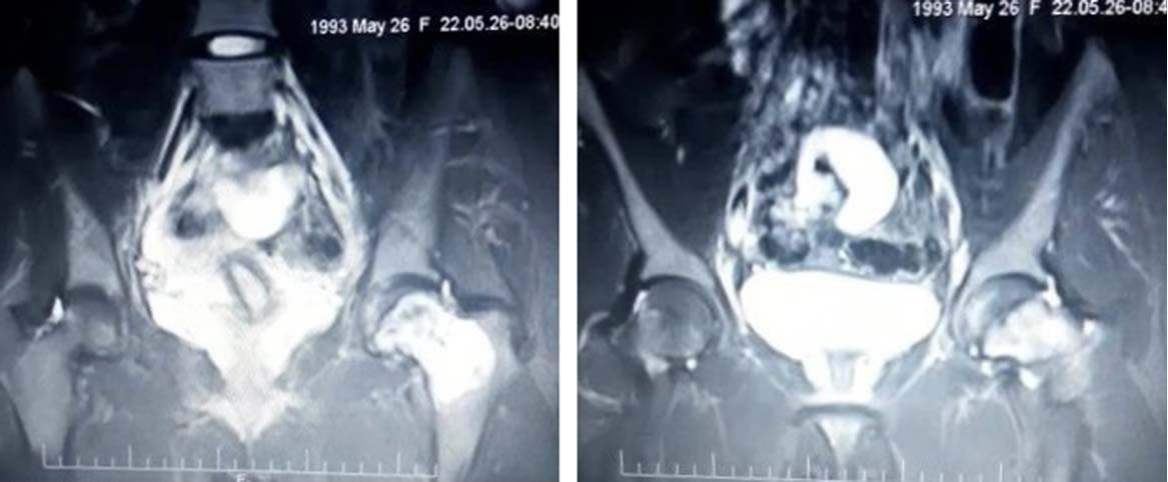
T1 weighted MRI showing osteolytic lesion with breach in the cortex.

Anteroposterior (AP) X-ray (Fig. 1) showed an intracapsular femoral neck fracture with an underlying osteolytic lesion surrounded by smooth sclerotic margins in the inferior part of the left neck of the femur, involving more than 50% of the neck diameter. MRI of the left hip (Fig. 2) showed an eccentric osteolytic lesion that takes most of the neck of the left femur which measured 64×63×25 mm, with a breach in cortex coupled with a sharp zone of transition and periosteal reaction.

In the operation room and under epidural anesthesia, the surgery was performed by a fifth-year resident surgeon under the supervisor’s view. A prophylactic cefazolin (2 g) was given before the surgery. We applied an orthopedic traction table and did a lateral 15 cm incision starting from the tip of the greater trochanter. Going between the Tensor Fascia Lata and Vastus Lateral’s until approaching the lateral edge of the top of the femur. Reaming of the neck of the femur was done up to 18 mm in diameter toward the greater trochanter and 14 mm for 3 cm toward the head of the femur. Curettage of the lesion was done, and the specimen was sent for histopathology. A total of 6 cm free fibula was placed into the cored-out neck of the femur as a snug fit with fixation by Dynamic hip screw (DHS), an 85 mm hip screw with four cortical distal screws.

After the surgery, the patient was transferred to the Orthopedic ward and discharged after 2 days with a prescription of Apixaban (2.5 mg twice a day) for 8 weeks.

**Figure 3:**
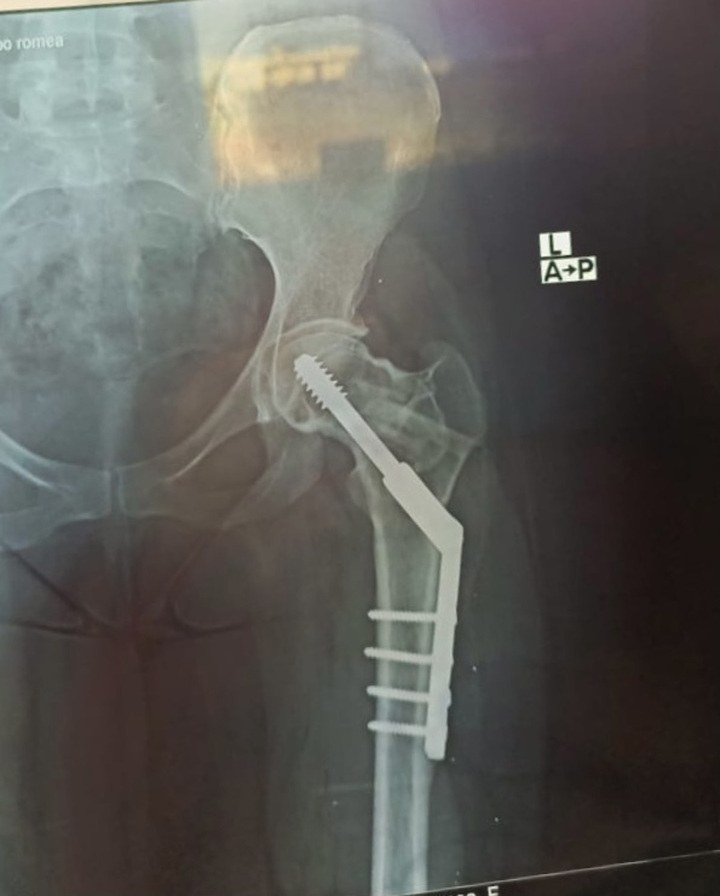
Post 6 months AP X-ray.

The postoperative period was uneventful. Weight-bearing was prohibited for 8 weeks, and full-weight bearing was allowed after 3 months. The fracture united and the patient was recovered and restored all movement after 6 months of follow-up (Fig. 3).

## Discussion

NOF predominantly occurs in the lower extremities, especially around the knee. Only very few cases of NOFs located in the femoral neck have been reported^8^, which were treated with curettage and neovascularized bone grafting. We are discussing a difficult case of NOF of intracapsular femoral neck treated with free fibula graft and stabilized by DHS, which proved to be a successful treatment modality under these conditions.

Most patients are asymptomatic, presenting as an incidental radiological finding of a lucent lesion, with the margins ranging from being densely sclerotic or scalloped to being hazy and indistinct^4^. The cortex may be thinned, and in some cases, it is expanded. NOF may rarely result in pathologic fractures. Surgical intervention is considered when the lesion leads to pathological fracture or there is a potential risk for the same. Arata *et al*.^3^ described that a lesion involving more than 50% of the transverse diameter or measuring 33 mm carries the risk of pathologic fracture.

Our patient was asymptomatic for months except for dull pain in the hip, eventually, she came with a pathologic fracture. For this challenging situation with a paucity of options, the patient was planned for curettage, fibular graft, and DHS stabilization, this option was chosen because of the emergent situation of intracapsular femoral neck fracture in young patient. This not only provides mechanical cortical support but also has regenerative potential^9^.

A fibular graft is a preferred source in such cases, as it provides suitable length and cortical support^10^.

The rich vascularity and biological potential of this graft allow callus formation from the deliberately exposed cortex and the periosteal cambial layer of the cephalad end of the graft to the cancellous bone and the remaining subchondral bone^11^.

The treatment of choice in such cases in young patients is curettage with vascularized fibula graft with or without fixation^12^, applying such treatment required a surgeon with experience in microscopic surgery and well-equipped center, the fixation can be done by cannulated screws or DHS, in the older patients, Total Hip Replacement could be considered.

We choose to stabilize the fracture by DHS because of the proven efficacy in these types of fractures and such younger ages, in which results were very gratifying with complete recovery in 6 months follow-up.

In such rare and challenging cases knowing the unique vasculature of neck of the femur, the risk of failure of the fixation is higher which leads to non-union, especially in young patients where Total Hip Arthroplasty is not preferred, that is why vascularized fibula transfer is the treatment of choice. This study demonstrates that the surgical treatment with free fibular bone graft for pathological femoral neck fractures and underlying NOFs allows a quick return to mobility and can achieve excellent postoperative recovery.

## Conclusion

Knowing that the vascularized fibular transfer bone grafting is the best treatment option in such cases with wide curettage, this case highlights the effectiveness of free fibular graft alongside hardware can be used in large lesions or pathologic fractures, in the situation of lacking microscopic surgery techniques, and they have regenerative potential and provides better mechanical stability.

Conducting further studies would be crucial to determine the effectiveness and adequacy of this treatment option.

## Ethical approval

Written informed consent was obtained from the patient for publication of this case report and accompanying images, in line with local ethical approval requirements and in accordance with the Helsinki Declaration.

## Consent

Written informed consent was obtained from the patient for publication of this case report and accompanying images. A copy of the written consent is available for review by the Editor-in-Chief of this journal on request.

## Sources of funding

This research did not receive any specific grant from funding agencies in the public, commercial, or not-for-profit sectors.

## Author contribution

M.A.M.: writes the manuscript, literature search, treat and follow-up the patient, and submitted the article; H.M. and O.M.A.: writes the manuscript, literature search, treat, and follow-up the patient; A.E.A.: manuscript correction, treat and follow-up the patient literature search, and supervisor of the case.

## Conflicts of interest disclosure

There are no conflicts of interest.

## Research registration unique identifying number (UIN)

1. Name of the registry: open science framework.
2. Unique identifying number or registration ID: 0009-0001-6528-4812.
3. Hyperlink to your specific registration (must be publicly accessible and will be checked): https://osf.io/hf3g4/?view_only=9687d8e28f2f4f65b0c48e6662e4bbf3

## Guarantor

The corresponding author is the guarantor of this manuscript.

## Data availability statement

Not available.

## Provenance and peer review

Not commissioned, externally peer reviewed.

## References

[1] Goldin A Muzykewicz DA Dwek J. The aetiology of the non-ossifying fibroma of the distal femur and its relationship to the surrounding soft tissues. J Child Orthop 2017;11:373–379. 29081852 10.1302/1863-2548.11.170068 PMC5643931

[2] Mirra JM. Bone tumors. Lea and Febiger; 1989;1:692–719.

[3] Arata MA Peterson HA Dahlin DC. Pathological fractures through non-ossifying fibromas. review of the Mayo Clinic experience. J Bone Joint Surg Am 1981;63:980–988. 7240338

[4] Ritschl P Karnel F Hajek P. Fibrous metaphyseal defects–determination of their origin and natural history using a radiomorphological study. Skeletal Radiol 1988;17:8–15. 3358140 10.1007/BF00361448

[5] Betsy M Kupersmith LM Springfield DS. Metaphyseal fibrous defects. J Am Acad Orthop Surg 2004;12:89–95. 15089082 10.5435/00124635-200403000-00004

[6] Havitçioğlu H Biçen Ç Hapa O. Treatment of intracapsular femoral neck lesions: aggressive or conservative surgery? Musculoskelet Surg 2014;98:251–254. 23263835 10.1007/s12306-012-0236-x

[7] Agha RA Franchi T Sohrab C. The SCARE 2020 guideline: updating consensus Surgical Case Report (SCARE) guidelines. Int J Surg 2020;84:226–230. 33181358 10.1016/j.ijsu.2020.10.034

[8] Kumar R Madewell JE Lindell MM. Fibrous lesions of bones. Radiographics 1990;10:237–256. 2158129 10.1148/radiographics.10.2.2158129

[9] Gilbert A. Free vascularized bone grafts. Int Surg 1981;66:27–31. 7019124

[10] Hase T Miki T. Autogenous bone marrow graft to non-ossifying fibroma with a pathologic fracture. Arch Orthop Trauma Surg 2000;120:458–459. 10968540 10.1007/s004029900100

[11] Korompilias AV Lykissas MG Beris AE. Vascularised fibular graft in the management of femoral head osteonecrosis: 20 years later. J Bone Joint Surg Br 2009;91:287–293. 19258601 10.1302/0301-620X.91B3.21846

[12] Parwaz M Chaudhary T Bansal S. Possible novel treatment modality for non-ossifying fibroma neck of femur. Indian J Plast Surg Dec 2020;53:442–446. 10.1055/s-0040-1716435 PMC7775256 33402782

